# Temporal trends in open thoracic aortic surgery in Sweden over 20 years: a nationwide registry-based study

**DOI:** 10.1016/j.lanepe.2026.101627

**Published:** 2026-02-21

**Authors:** Jenny Backes, Nadia Sandström, Maja Eriksson Östman, Daniel Robert Smith, Anders Jeppsson, Anna Jonsson Holmdahl, Örjan Friberg

**Affiliations:** a School of Medical Sciences, Faculty of Medicine and Health, Örebro University, Örebro, Sweden; b Department of Cardiothoracic and Vascular Surgery, Örebro University Hospital, Örebro, Sweden; c Department of Cardiology, Örebro University Hospital, Örebro, Sweden; d Clinical Epidemiology and Biostatistics, School of Medical Sciences, Faculty of Medicine and Health, Örebro University, Örebro, Sweden; e Department of Molecular and Clinical Medicine, Institute of Medicine, Sahlgrenska Academy, University of Gothenburg, Gothenburg, Sweden; f Department of Cardiothoracic Surgery, Sahlgrenska University Hospital, Gothenburg, Sweden; g Department of Public Health and Clinical Medicine, Umeå University, Umeå, Sweden; h Department of Cardiothoracic Surgery, Umeå University Hospital, Umeå, Sweden

**Keywords:** Thoracic aortic surgery, Aortic aneurysm, Aortic dissection, Temporal trends, Population-based study, Postoperative mortality, Stroke, Sweden, Nationwide registry

## Abstract

**Background:**

There is a paucity of contemporary population-based studies on temporal trends in incidence and early complications after open thoracic aortic surgery. This study aimed to assess temporal trends in incidence and early complications of open aortic surgery for ascending aortic aneurysm or dissection in Sweden.

**Methods:**

All open thoracic aortic operations for aortic aneurysm or dissection involving the ascending aorta, in Sweden from 2001 to 2020 were included in a nationwide, population-based, observational cohort study. Individual patient data were collected from the SWEDEHEART registry, the National Patient Registry, and the National Cause of Death Register. The incidences of surgery, early postoperative mortality, and stroke rates over time were compared using generalized additive models, separately for patients operated for aneurysms and dissections.

**Findings:**

A total of 10,089 procedures in 9829 patients were included. In total, 6429/10,089 operations were performed for aneurysms (63.7%) and 3660/10,089 for dissections (36.3%). The annual incidence of open thoracic aortic operations increased from 26.7 per million inhabitants in 2001 to 64.0 in 2020 (p for trend <0.001). Crude 30-day mortality after first-time surgery was 2.4% (95% confidence interval (CI) 2.0–2.9) for aneurysms and 14.6% (95% CI 13.4–15.8) for dissections. The age- and sex-adjusted 30-day mortality risk for first-time surgery for aneurysm decreased over time (adjusted odds ratio (aOR) 0.33, 95% CI 0.14–0.77) while stroke risk showed a tendency toward decrease (aOR 0.42, 95% CI 0.17–1.06). For first-time surgery for dissections, adjusted 30-day mortality risk decreased significantly (aOR 0.40, 95% CI 0.25–0.64), whereas stroke risk did not change (aOR 1.05, 95% CI 0.64–1.72).

**Interpretation:**

The incidence of open thoracic aortic surgery in Sweden for aneurysm and dissection involving the ascending aorta, more than doubled over two decades. Survival after aneurysm and dissection surgery improved during the study period.

**Funding:**

This study was supported by the Örebro University Hospital Research Foundation and Nyckelfonden Research Foundation.

**Research in context**

**Evidence before this study**

We searched PubMed for nationwide or population-based studies on temporal trends in aortic surgery. Search terms included “aortic surgery”, “aortic aneurysm”, “aortic dissection”, “nationwide”, “population-based”, and “temporal trends”. Reference lists of relevant papers were also reviewed. Most identified studies lacked complete national coverage, excluded either aneurysms or dissections, were limited in time, or focused on earlier time periods. Despite changes in surgical practice and clinical management over recent decades, no recent nationwide study has comprehensively examined long-term trends in incidence, indications, and outcomes of aortic surgery.

**Added value of this study**

This nationwide observational study provides a comprehensive analysis of open thoracic aortic surgery in Sweden over two decades. The incidence of surgery more than doubled between 2001 and 2020, mainly driven by increased procedures for aneurysms. During this period, surgical mortality declined substantially. One-year mortality remained higher after surgery for dissection than after surgery for aneurysm, although the excess risk was largely confined to the first 30 postoperative days. These findings provide contemporary, population-based benchmarks for outcomes after open aortic surgery.

**Implications of all the available evidence**

The improved outcomes observed over time likely reflect advances in surgical and perioperative management, as well as evolving clinical decision-making and guideline implementation. These results should be considered when balancing the risks and benefits of surgery in this often complex and high-risk patient population.

## Introduction

Thoracic aortic aneurysms are often asymptomatic and incidentally detected during imaging for unrelated conditions. In contrast, an aortic dissection is an acute event caused by an intimal tear, potentially leading to aortic rupture, organ malperfusion, cardiac tamponade, and aortic valve regurgitation. If left untreated, the acute dissection may progress to a chronic dissection. The presence of an aneurysm, particularly in patients with additional risk factors for dissection, significantly increases the risk of dissection and rupture.^1^^,^^2^

Aneurysms and dissections located in the ascending aorta and/or the aortic arch are typically managed via open surgical repair involving prosthetic graft replacement. However, aortic surgery is performed not only on patients with aneurysms and dissections but also occasionally on patients with other conditions, such as aortic valve endocarditis, aortic ulcerations, coarctation, and pseudoaneurysms. The underlying disease significantly impacts perioperative and short-term mortality, with markedly lower mortality and complication rates for elective thoracic aortic aneurysm surgery than surgery for acute aortic dissection or acute endocarditis.^3^, ^4^, ^5^

A nationwide observational cohort study on the prevalence, incidence, and outcome of thoracic aneurysms and dissections in Sweden from 1987 to 2002 was published in 2006.^6^ The study showed a large increase in surgical procedures for both thoracic aortic aneurysms and dissections in both men and women. Survival improved significantly in both men and women during that period. It is unclear whether the incidence of thoracic aortic surgery and survival rates following surgery have continued to increase since the end of the study inclusion period in 2002. Thus, the present nationwide population-based study aimed to describe temporal trends in the incidence and early complication rates for open thoracic aortic surgery for aortic aneurysm or dissection involving the ascending aorta, with or without concomitant aortic arch surgery, in Sweden over a 20-year period starting 2001.

## Methods

### General

This study was conducted according to the 1975 Declaration of Helsinki and approved by the Swedish Ethical Review Authority (registration number 2023-01921-01; approved April 26, 2023). The authority waived the need for individual patient consent due to the retrospective study design. The Strengthening the Reporting of Observational Studies in Epidemiology (STROBE) guidelines were adhered to in the writing of this manuscript.^7^

### Procedures and study design

All open operations for aortic aneurysm or dissection involving the ascending aorta, with or without concomitant aortic arch surgery, and performed with extracorporeal circulation from January 2001 to December 2020 in patients over 18 years old were included in this nationwide observational population-based cohort study, based on prospectively collected data. The operations were identified in the Swedish Cardiac Surgery Registry,^8^ which is part of the Swedish Web-system for Enhancement and Development of Evidence-based care in Heart disease Evaluated According to Recommended Therapies (SWEDEHEART) registry.^9^ Operations were identified by procedural codes from the Swedish version of the Nomesco Classification of Surgical Procedures.^10^ A detailed description of the procedure codes is presented in Supplementary Table S1.

**Fig. 1:**
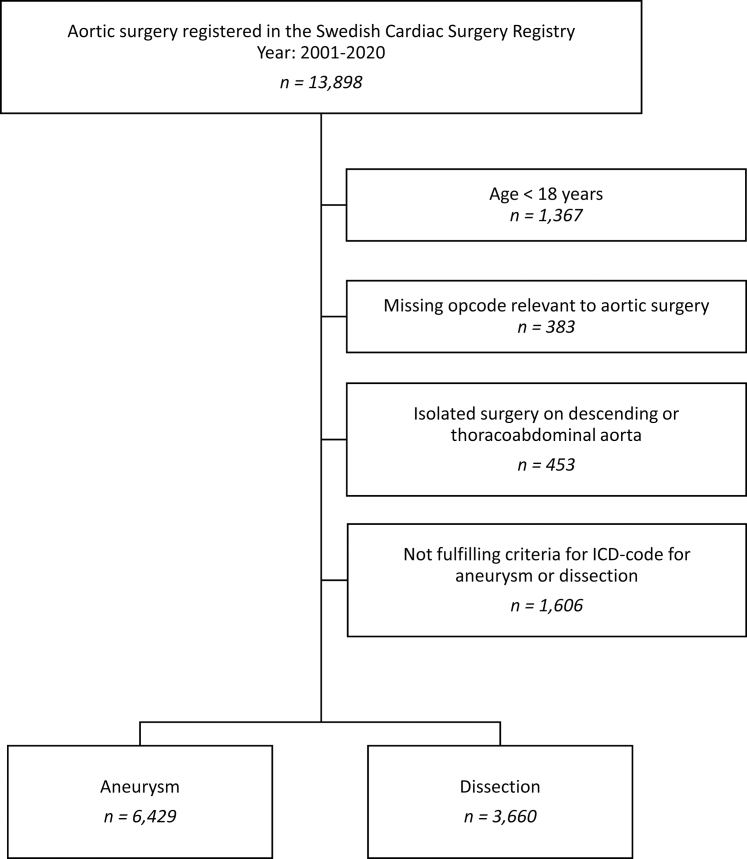
Flowchart of the study population with subgroup stratification.

The operations were divided into subgroups based on aetiology. Only aortic aneurysms or dissections were included in the further analyses. Diagnostic codes from the tenth revision of the International Classification of Diseases (ICD-10) were used to define the subgroups. Aneurysms were defined as either of I71.2, I71.6 or I71.9 but not fulfilling the criteria for dissection. Acute and chronic dissections were defined as either of I71.0, I71.1, I71.5 or I71.8. Ruptured aortic aneurysms and post-dissection aneurysms were included in the dissection group. Reoperations on the aorta during the follow-up period were also included. A flowchart is presented in Fig. 1. Data from SWEDEHEART were merged with information from the National Patient Register^11^ and the National Cause of Death Register^12^ at the time of data extraction from SWEDEHEART using the unique personal identification number provided to all Swedish citizens at birth or immigration.^13^ The gradual inclusion of additional variables in the Swedish Cardiac Surgery registry led to an increase in available information over the study period.

### Definitions

The following definitions from the Cardiac Surgery Registry were used. Diabetes included both insulin-, oral- and diet-treated diabetes. Stroke was defined as a sudden onset of circulation-related central neurological injury occurring during or after surgery, with symptoms lasting >72 h, and identified before discharge from the cardiac surgery department. Confirmation with imaging was not necessary. A reoperation was defined as a previous open heart or aortic surgery reported in the Cardiac Surgery Registry.

### Data sources

Data on the aortic surgery procedures, including preoperative status and in-hospital complications, were retrieved from SWEDEHEART, which contains detailed information on all cardiac operations in Sweden since 1992, with a coverage of >99% during the study period.^8^ Additional information about baseline variables, diagnoses, and events was collected from the National Patient Register, which has full coverage of diagnoses from all hospital admissions in Sweden since 1987, with a validity of 85–95%.^11^ Hospitalisations were identified and classified according to the ICD-10. Mortality data were collected from the national Cause of Death Register, which holds information on the date and cause of death of all deceased Swedish citizens according to the ICD-10 since 1961.^12^ Population data were obtained from the Total Population Registry held by Statistics Sweden (SCB).^14^

### Statistical analyses

Continuous variables were summarised using the mean with standard deviation (SD). Categorical variables were summarised using frequencies and percentages.

Crude annual incidence of operations, stratified by diagnosis and sex, was calculated as the number of operations performed each year divided by the national population at the end of that year. Crude event rates for 30-day mortality and perioperative stroke, also stratified by diagnosis and sex, were calculated as the number of events divided by the total number of operations performed. These outcomes were treated as binary variables, and 95% confidence intervals were estimated using exact binomial methods. Confidence intervals for absolute differences in proportions between time periods were estimated using exact binomial methods.

Generalized additive models (GAMs) assuming the negative binomial distribution and a logarithmic link function were used to model the incidence of operations over time. The response variable was the number of operations in a given year, but by including the logarithm of the total number of operations in each year as an offset term (coefficient constrained to 1), we effectively modelled the incidence rate. We also used GAMs to model the binary outcomes 30-day mortality and perioperative stroke, assuming the binomial distribution with a logit link function. Separate GAMs were fitted for each combination of diagnostic group (aneurysm, dissection) and sex (women, men, all) using restricted maximum likelihood (REML) as the smoothing parameter method. All smooth terms were modelled using thin plate regression splines, a basis dimension of k = 10 was specified for the univariate spline components. Time (calendar year) was modelled flexibly using a thin plate spline.^15^ To obtain adjusted effects, age was also modelled using a thin plate spline, and sex was included as a dummy variable for the data subset, which included both men and women. For 30-day mortality and perioperative stroke, relative effects for each calendar year (compared with the year 2001 as the reference) are presented as odds ratios (ORs), with confidence intervals (CIs) adjusted for simultaneous inference using the Dunnett method.

Mortality data were complete. For perioperative stroke, fewer than 2% of observations were missing. Given the low proportion of missing data, analyses were performed using a complete-case approach. Multiple imputation was not undertaken, as differences between complete-case analysis and multiple imputation are generally minimal when the proportion of missing data is below 5%.^16^

We performed a survival analysis of time from operation to all-cause mortality. Follow-up time was calculated from the date of surgery to the date of death or censoring (December 31, 2022). For patients with several recorded operations in the dataset, only the first operation was considered. The results can thus only be interpreted in the context of first-time operations. Cumulative survival probabilities were estimated using the Kaplan–Meier method. A sensitivity analysis excluding the first COVID year was performed.

The significance threshold was set at 0.05; accordingly, uncertainty was quantified using 95% CIs. All statistical analyses were performed using R version 4.3.3 in RStudio version 2024.9.1.394, and included use of the “tidyverse,” “mgcv,” “marginaleffects,” “survival,” and “emmeans” packages.^15^^,^^17^, ^18^, ^19^, ^20^, ^21^

### Role of funding source

The funding sponsor had no role in the study design; collection, management, analysis, or interpretation of data; in writing of the manuscript, or the decision to submit for publication.

## Results

### Patients/study cohort

There were 10,089 individual operations with relevant procedural codes identified in the Cardiac Surgery Registry. Baseline characteristics are presented in Table 1. The overall mean age of the patients was 61.6 years, with more than two-thirds being male. Mean patient age increased significantly during the study period (Supplementary Figure S1).

**Table 1:** Baseline characteristics.

Characteristic	All procedures n = 10,089	Aneurysm n = 6429	Dissection n = 3660
Age (years), mean (SD)	61.6 (12.5)	61.3 (12.8)	62.1 (11.9)
Age category (years), n (%)			
≤64	5262 (52.2)	3334 (51.9)	1928 (52.7)
65–74	3490 (34.6)	2288 (35.6)	1202 (32.8)
≥75	1337 (13.3)	807 (12.6)	530 (14.5)
Sex, n (%)			
Female	3091 (30.6)	1870 (29.1)	1221 (33.4)
Creatinine clearance (ml/min), mean (SD)	92.5 (35.3)	95.7 (35.1)	85.9 (34.8)
Unknown, n	1092	399	693
Diabetes, n (%)	477 (4.9)	335 (5.3)	142 (4.1)
Unknown, n	309	142	167
CHA_2_DS_2_-VASc, mean (SD)	2.7 (1.5)	2.7 (1.4)	2.6 (1.5)
Previous stroke, n (%)	561 (8.5)	302 (7.0)	259 (11.3)
Unknown, n	3485	2114	1371

Aortic aneurysm was the indication for surgery for 6429/10,089 (63.7%) operations and aortic dissection for 3660/10,089 (36.3%) operations. The operations involved total arch replacements in 546/10,089 (5.4%) of the cases. The extent of the aortic replacement is reported in Supplementary Tables S2 and S3.

The proportion of aneurysm surgery in the cohort increased from 59.3% during the first 5-year period (2001–2005) to 65.2% during the last 5-year period (2016–2020), difference 5.9% (95% CI 2.9–8.8). The proportion of surgery for dissection decreased correspondingly between the first and last 5-year period (40.7% vs. 34.8%). A total of 831 of the 10,089 operations (8.2%) were reoperations. Baseline characteristics and outcomes stratified by 5-year intervals during the study period are summarised for all procedures in Table 2, and for surgery for aneurysm and dissection in Supplementary Tables S4 and S5, respectively.

**Table 2:** Baseline characteristics and outcomes stratified by 5-year intervals during the study period (all procedures).

Time period	Overall n = 10,089	2001–2005 n = 1582	2006–2010 n = 2286	2011–2015 n = 2816	2016–2020 n = 3405
Age (years), mean (SD)	61.6 (12.5)	60.5 (12.8)	61.1 (12.1)	61.8 (12.4)	62.3 (12.6)
Age category (years), n (%)					
≤64	5262 (52.2)	902 (57.0)	1264 (55.3)	1436 (51.0)	1660 (48.8)
65–74	3490 (34.6)	492 (31.1)	735 (32.2)	1023 (36.3)	1240 (36.4)
≥75	1337 (13.3)	188 (11.9)	287 (12.6)	357 (12.7)	505 (14.8)
Sex, n (%)					
Female	3091 (30.6)	463 (29.3)	711 (31.1)	893 (31.7)	1024 (30.1)
Creatinine clearance (ml/min), mean (SD)	92.5 (35.3)	84.9 (33.7)	93.5 (35.4)	94.3 (35.8)	93.3 (35.1)
Unknown, n	1092	334	394	259	105
Diabetes, n (%)	477 (4.9)	46 (3.1)	94 (4.5)	148 (5.3)	189 (5.6)
Unknown, n	309	87	185	29	8
CHA_2_DS_2_-VASc, mean (SD)	2.7 (1.5)	1.6 (1.2)	2.2 (1.2)	2.7 (1.3)	3.4 (1.5)
Diagnosis group, n (%)					
Aneurysm	6429 (63.7)	938 (59.3)	1446 (63.3)	1826 (64.8)	2219 (65.2)
Dissection	3660 (36.3)	644 (40.7)	840 (36.7)	990 (35.2)	1186 (34.8)
30-day mortality (first-time operations only), n (%)	634 (6.8)	161 (10.7)	151 (7.1)	156 (6.1)	166 (5.4)
Perioperative stroke, n (%)	713 (7.2)	133 (9.1)	140 (6.3)	187 (6.7)	253 (7.4)
Unknown, n	196	116	59	17	4

### Incidence of thoracic aortic surgery over time

**Fig. 2:**
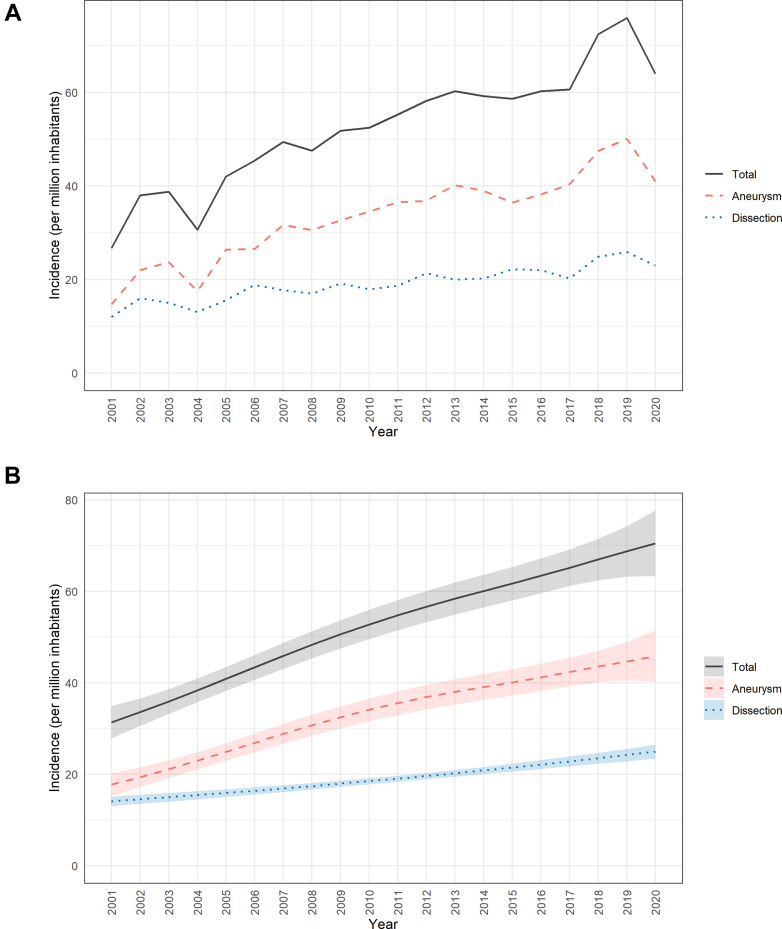
a. Annual incidence of surgery for aneurysm, dissection, and total per million inhabitants in Sweden, 2001–2020. Lines represent observed incidence by diagnosis group. b. Estimated annual incidence of surgery for aneurysm, dissection, and total per million inhabitants in Sweden, 2001–2020. Estimates are from generalized additive models. Shaded areas represent 95% confidence bands.

The actual annual incidence of open thoracic aortic surgery involving the ascending aorta with or without concomitant aortic arch surgery in Sweden more than doubled from 2001 to 2019, from 26.7 per million inhabitants in 2001 to 75.9 per million in 2019 (p for trend <0.001), with a decrease to 64.0 per million in 2020 (the first year of the COVID-19 pandemic) (Fig. 2a). There was a threefold absolute increase in surgery for aortic aneurysm from 14.7 per million in 2001 to 50.1 per million in 2019 (p for trend <0.001), with a decrease to 40.9 per million in 2020.

**Fig. 3:**
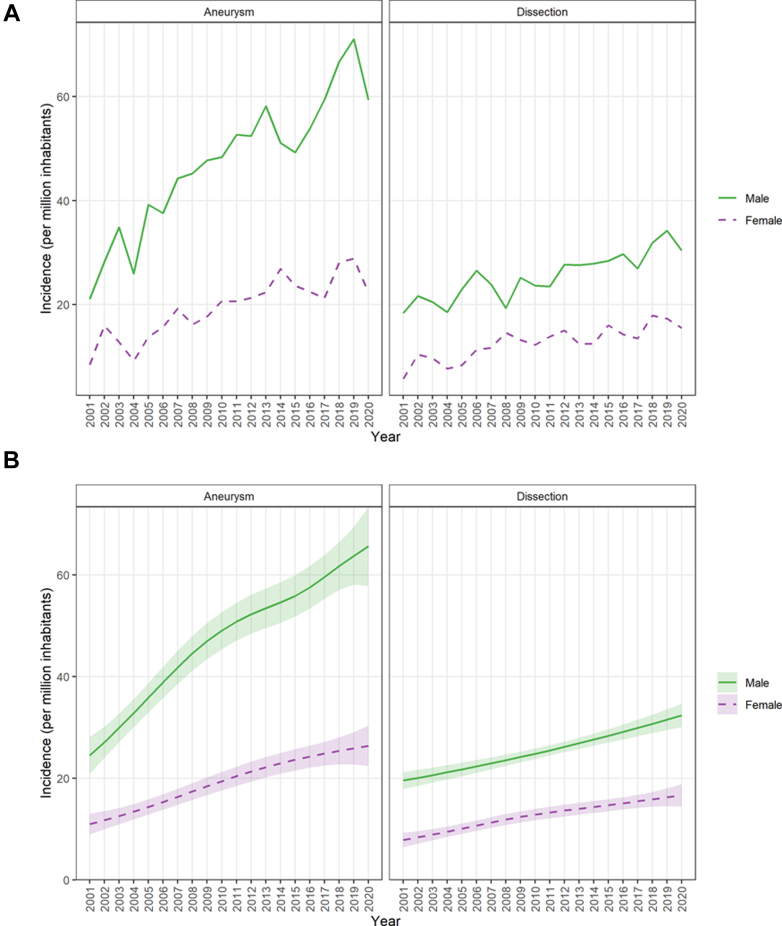
a. Annual incidence of surgery for aneurysm and dissection per million inhabitants in Sweden by sex, 2001–2020. Lines show observed incidence by diagnosis group and sex. b. Estimated annual incidence of surgery for aneurysm and dissection per million inhabitants in Sweden by sex, 2001–2020. Estimates are from generalized additive models. Shaded areas represent 95% confidence bands.

The incidence of surgery for thoracic aortic dissection doubled from 2001 to 2019, from 12.0 per million in 2001 to 25.9 per million in 2019 (p for trend <0.001), with a reduction to 23.0 per million in 2020. Sex-specific incidence curves for aneurysms and dissections are presented in Fig. 3a. The incidences were consistently higher in men than in women for both aneurysms and dissections. Corresponding model-based estimates are presented in Figs. 2b and 3b.

### Thirty-day mortality

**Fig. 4:**
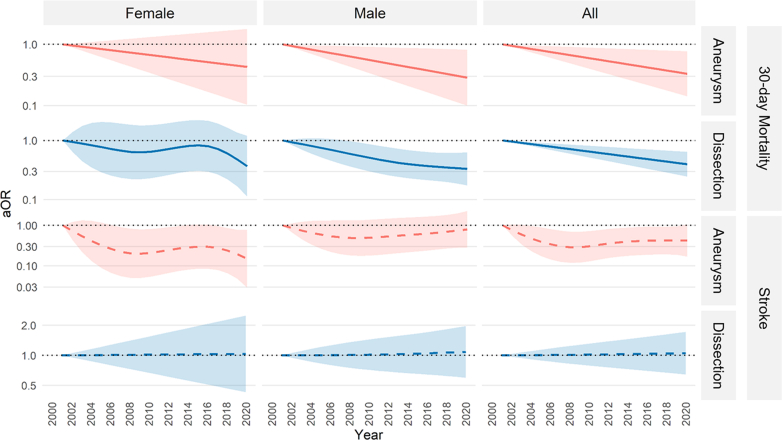
Odds ratios for 30-day mortality and perioperative stroke from generalized additive models, stratified by diagnosis (aneurysm or dissection) and sex, and adjusted for age and sex where applicable. First-time operations only. Shaded areas indicate 95% confidence intervals. Reference year: 2001 (dotted line). aOR, adjusted odds ratio.

The crude 30-day mortality after surgery for all first-time operations (reoperations excluded) was 2.4% (95% CI 2.0–2.9) for aneurysms and 14.6% (95% CI 13.4–15.8) for dissections. Crude 30-day mortality for patients who had previously undergone a cardiac or aortic surgery procedure was 4.1% (95% CI 2.6–6.2) for aneurysms and 14.5% (95% CI 10.7–19.0) for dissections. Crude 30-day mortality for first-time operations decreased over time in patients operated for aneurysm, from 3.9% (95% CI 2.8–5.4) during the first 5-year period (2001–2005) to 1.8% (95% CI 1.3–2.5) during the last 5-year period (2016–2020). After adjusting for age and sex, 30-day mortality risk for first-time operations for aneurysms was significantly lower in 2020 than in 2001 (adjusted OR (aOR) 0.33, 95% CI 0.14–0.77). The decrease in adjusted 30-day mortality risk was statistically significant in men (aOR 0.29, 95% CI 0.10–0.82) but not in women (aOR 0.43, 95% CI 0.10–1.79). Fig. 4 and Supplementary Table S6 show aORs over time.

Crude 30-day mortality after first-time operations for dissection decreased from 20.5% (95% CI 17.3–23.9) to 12.0% (95% CI 10.2–14.1) from the first to the last 5-year period. After adjusting for age and sex, 30-day mortality risk for first-time operations for dissection was significantly lower in 2020 than in 2001 (aOR 0.40, 95% CI 0.25–0.64). The decrease in adjusted 30-day mortality risk was statistically significant in men (aOR 0.33, 95% CI 0.17–0.63) but not in women (aOR 0.37, 95% CI 0.11–1.20). Fig. 4 and Supplementary Table S7 show aORs over time. Thirty-day mortality in relation to age is presented in Supplementary Figures S2 and S3.

### Perioperative stroke

The crude perioperative stroke rate for first-time operations was 3.3% (95% CI 2.8–3.8) for aneurysms and 14.3% (95% CI 13.1–15.5) for dissections. The crude stroke rate for patients who had previously undergone a cardiac or aortic surgery procedure was 5.7% (95% CI 3.9–8.0) for aneurysms and 9.0% (95% CI 5.9–12.9) for dissections. The crude stroke rate for first-time operations for aneurysms was 5.1% (95% CI 3.7–6.8) in the first and 3.5% (95% CI 2.8–4.4) in the last 5-year period. The age- and sex adjusted stroke risk for first-time operations for aneurysms tended toward a decrease between 2001 and 2020 (aOR 0.42, 95% CI 0.17–1.06). When data for male and female patients were analysed separately, a significant decrease in adjusted stroke risk in women (aOR 0.15, 95% CI 0.03–0.79) but not in men (aOR 0.80, 95% CI 0.28–2.27) was noted. Fig. 4 and Supplementary Table S8 show aORs over time.

The crude stroke rate in patients who underwent a first-time operation for dissection was 15.6% (95% CI 12.7–18.9) in the first and 14.4% (95% CI 12.4–16.6) in the last 5-year period. The adjusted stroke risk following first-time surgery for aortic dissection did not differ significantly between 2001 and 2020 overall (aOR 1.05, 95% CI 0.64–1.72), in men (aOR 1.08, 95% CI 0.60–1.97), or in women (aOR 1.03, 95% CI 0.43–2.50). Fig. 4 and Supplementary Table S9 show aORs over time. Stroke incidence in relation to age is presented in Supplementary Figures S2 and S3. The sensitivity analysis of mortality and stroke excluding the first year of the COVID pandemic, supported the main results, Supplementary Figure S4.

### One-year overall survival

**Fig. 5:**
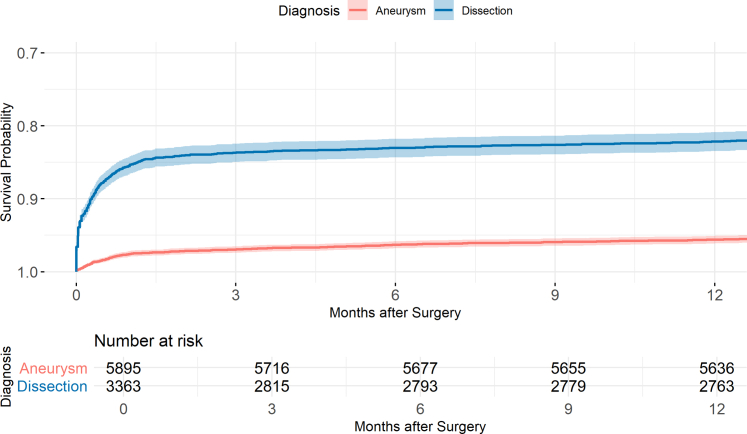
Kaplan–Meier survival for the first year following surgery for aneurysm and dissection, first-time-operations only.

One-year survival following first-time surgery for aneurysm was 95.6% (95% CI 95.1–96.1) vs. 82.2% (95% CI 80.9–83.5) for dissection. The Kaplan–Meier survival curves diverged early, reflecting the marked difference in 30-day mortality between the two groups. Thereafter, the curves remained approximately parallel, indicating similar survival beyond the initial postoperative period. A Kaplan–Meier survival curve for the first year postoperatively is presented in Fig. 5. Hospital stays over time are presented in Supplementary Tables S10 and S11.

## Discussion

The main findings of this large, nationwide population-based study over a 20-year period were as follows: (1) the incidence of open aortic surgery more than doubled between 2001 and 2020, (2) the largest increase in incidence was observed in surgery for aortic aneurysms, and (3) early mortality significantly decreased over the study period, after both elective surgery for aneurysm and acute surgery for aortic dissection, even though the mean patient age increased during the study period.

In 2007, Olsson et al. published a nationwide population-based cohort study on the prevalence, incidence, and outcome of thoracic aneurysms and dissections from 1987 to 2002 in Sweden.^8^ The study showed a sevenfold increase in the incidence of surgery for thoracic aortic aneurysm or dissection, in addition to markedly improved survival rates over the study period.^6^ Resuming from the year in which the above study ended, the present study showed that the incidence of open surgery on the thoracic aorta continued to increase, although at a slower pace, over the subsequent 20 years. The most pronounced increase was observed in procedures for aortic aneurysm, although significant increases were also observed in aortic dissection surgery. Population-based studies describing temporal trends in thoracic aortic surgery are rare; however, increased incidence of aneurysm and dissection surgery has been reported in single-centre studies, register-based studies, and regional studies.^6^^,^^22^, ^23^, ^24^ Several factors may contribute to the continued rise in surgery for thoracic aortic aneurysm. It is unlikely that the actual prevalence of aneurysmal disease has increased substantially. Rather, improved access to imaging techniques such as computed tomography and echocardiography has likely led to a higher rate of incidental detection of aneurysms. Additionally, there is likely a growing tendency to offer prophylactic surgery to older patients, which may reflect both evolving clinical guidelines^2^^,^^25^ and improved perioperative outcomes in older patients.^22^ In the present study, mean patient age increased during the study period, especially in patients who underwent surgery for dissection.

The present study period includes the first year of the COVID-19 pandemic, wherein a decline in cardiac surgery procedures has been reported.^26^ A similar decrease was observed in the present study. It is likely that the overall increase in surgical incidence would have been even more pronounced in the absence of the pandemic. It could be argued that the year 2020 should have been excluded from trend analyses; however, we chose to retain it, as it represents the real-world conditions during the study period.

Despite the increase in prophylactic surgery for aortic aneurysm, the incidence of surgery for acute aortic dissection did not decrease. By treating aneurysms pre-emptively, some dissections should have been prevented. However, several explanations may account for this paradox. First, a larger proportion of patients with acute dissection may now be diagnosed and offered surgery due to improved imaging and heightened clinical awareness. Second, a greater number of older and frail patients may be undergoing surgery for dissection today than in previous decades. This is supported by the present study, where the mean age of patients undergoing surgery increased over time. Early mortality was markedly higher after surgery for dissection than for aneurysm, the difference occurred mainly within the first 30 days postoperatively.

The rates of mortality and stroke in this national study are comparable to previous reports. Zhu et al. reported a postoperative stroke rate of approximately 13–15% among patients undergoing surgery for aortic dissection at Stanford University Hospital, which is similar to our findings.^27^ In 2012, Williams et al. published outcomes after proximal aortic replacement in elective and urgent cases.^28^ The 30-day mortality was 3.4% for elective procedures and 15.4% for urgent procedures. Assuming that most urgent procedures were performed for aortic dissection and most elective procedures for aneurysm, these results are broadly consistent with the outcomes observed in our study.

The present study revealed a significant decline in 30-day mortality over time following both aortic aneurysm and dissection surgery, in accordance with other contemporary reports.^24^^,^^27^^,^^29^ This reduced mortality may be explained by improvements in patient selection, anaesthesia, and intensive care, as well as advances in surgical technique. The incidence of perioperative stroke tended to decrease for aneurysm surgery, which may be explained by the factors outlined above. However, the consistently higher stroke rate following surgery for dissection is likely attributable more to the complexity and acute nature of the underlying pathology than to the surgical procedure itself.

Strengths of the present study include the complete nationwide cohort and the use of validated registries. The large sample size, combined with advanced modelling techniques such as smoothing splines to allow for non-linear trends, provided high statistical power and precision in estimates. No patient was lost to follow-up. The study's limitations include its observational design, which inherently precludes any conclusions on causality due to the risk of undetected confounding and bias. ICD-10 coding has recognised limitations in accurately identifying the specific segmental anatomy and extent of thoracic aortic disease. Furthermore, the registries have limited granularity of surgical approach and details, operative techniques, extent of repair, cerebral protection strategies and perioperative management. Complete information about procedure urgency lacks in the database, prohibiting analyses whether improvements in outcome are driven by advances in surgical technique and perioperative care, or by earlier diagnosis and increased elective repair. Likewise, the exact definition of variables and the number of variables in the registries may have changed over time. Complete data on comorbidities and perioperative covariates were not available throughout the entire study period. Consequently, age and sex were the only confounders consistently recorded in the registries and were therefore used for adjustments. Data on ethnicity and race was not collected. Only open surgical procedures performed via sternotomy were included in the study. Until recently endovascular techniques have preferably been used for descending and arch pathology but may in the future play a significant role also in the treatment of the ascending aorta.

The scope of this study was to describe temporal trends in the incidence and early outcomes of surgery for aortic aneurysms and dissections without including detailed patient information and operative data, which would require further elaborate analyses. Hence, adjustments were made for only age and sex when reporting early mortality and stroke incidence over time. We acknowledge that several other factors influence early and late outcomes following cardiac surgery, such as (but not limited to) the patient's condition and the existence of comorbidities, localisation and extension of the aortic pathology, and surgical technique and approach, which may have varied over the study period. Further studies investigating these factors in large, well-defined, contemporary, nationwide study cohorts are warranted.

### Conclusions

The results of this population-based study demonstrate that the incidence of thoracic aortic surgery for aneurysms and dissections has continued to increase, and that clinical outcomes have continually improved over the 20-year study period.

## Contributors

JB: Conceptualisation, data curation, formal analysis, figures, writing original draft. NS: Data curation, writing–review and editing. MEÖ: Data curation, writing–review and editing. DRS: Data curation, supervision, formal analysis, figures, methodology, writing - review and editing. AJ: Methodology, supervision, writing–review and editing. AJH: Methodology, writing–review and editing. ÖF: Methodology, data curation, supervision, funding acquisition, writing–review and editing. JB and ÖF has directly accessed and verified the underlying data and were responsible for the decision to submit the manuscript.

## Data sharing statement

The data utilised in this study will be provided with publication upon reasonable request following approval by the relevant authorities.

## Declaration of interests

ÖF discloses personal payment for consultancy from AbbVie and speaker fee from SERB, both unrelated to the present study. AJ discloses personal payments for consultancy from AstraZeneca, Pharmacosmos, Werfen, LFB Biotechnologies and Novo Nordisk, and speaker's fees from Bayer and Boehringer-Ingelheim, all unrelated to the present work. None of the other authors report any conflict of interest.

## Appendix A Supplementary data

Supplement Final
