# Supplementary material for: Temporal trends in open thoracic aortic surgery in Sweden over 20 years: a nationwide registry-based study
Source: Lancet Reg Health Eur. 2026 Feb 21;64:101627. doi: 10.1016/j.lanepe.2026.101627 (PMC12938862; doi:10.1016/j.lanepe.2026.101627)

Supplementary to

Temporal trends in open thoracic aortic surgery in Sweden over 20 years: a nationwide registry-based study

Jenny Backes, Nadia Sandström, Maja Eriksson Östman, Daniel Robert Smith, Anders Jeppsson, Anna Jonsson Holmdahl, Örjan Friberg.

Content Page

Table S1. Included procedure codes from the Swedish procedure coding system 2

Table S2. Distribution of surgical procedures over time for aortic aneurysm 3

Table S3**. Distribution of surgical procedures over time for aortic** dissection **4**

**Table S4. Baseline characteristics and outcomes for aneurysms 5**

**Table S5. Baseline characteristics and outcomes for dissections 6**

**Table S6. Odds ratio for 30-days mortality after aneurysm surgery 7**

**Table S7. Odds ratio for 30-days mortality after dissection surgery 8**

**Table S8. Odds ratio for perioperative stroke after aneurysm surgery 9**

**Table S9. Odds ratio for perioperative stroke after dissection surgery 10**

**Table S10. Hospital length of stay after aortic aneurysm surgery 11**

**Table S11** Hospital length of stay after aortic dissection surgery 11

**Figure S1. Modelled mean age at surgery for aneurysm and dissection 12**

**Figure S2. Crude mortality and perioperative stroke incidence in patients operated
for aneurysm or dissection in different age groups 13**

**Figure S3. Odds ratios for 30-day mortality and perioperative stroke from
generalized additive models, stratified by diagnosis (aneurysm or dissection)
and age 14**

**Figure S4. Sensitivity analysis excluding the first year of the COVID pandemic.
Odds ratios for 30-day mortality and perioperative stroke from generalized
additive models, stratified by diagnosis (aneurysm or dissection) and sex. 15**

**Table S1. Included procedure codes from the Swedish procedure coding system.**

# **FCA Repair of ascending aorta**

| Code | Procedure Description |
| --- | --- |
| FCA 00 | Suture of ascending aorta |
| FCA 10 | Repair of ascending aorta by division and suture |
| FCA 20 | Reinforcement of ascending aorta with wrapping |
| FCA 30 | Partial resection and suture of ascending aorta |
| FCA 40 | Repair of ascending aorta using patch |
| FCA 45 | Percutaneous insertion of stent into ascending aorta *Includes: Of plain mesh stent and coated stent (stentgraft)* |
| FCA 50 | Resection and reconstruction of ascending aorta using tube graft |
| FCA 60 | Resection of aortic root and ascending aorta with reimplantation of coronary arteries and use of composite graft with mechanical valve prosthesis *Eponyms: Bentall-deBono, Cabrol* |
| FCA 70 | Resection of aortic root and ascending aorta with reimplantation of coronary arteries and use of biological valve prosthesis and tube graft  *Eponyms: Bentall-deBono, Cabrol* |
| FCA80 | Resection of the aortic root and ascending aorta with reimplantation of coronary arteries and preservation of the patient’s own aortic valve *Eponym: David* |
| FCA 83 | Removal of foreign body from ascending aorta |
| FCA 84 | Percutaneous removal of foreign body from ascending aorta |
| FCA 96 | Other repair of ascending aorta |

# **FCB Repair of aortic arch**

| Code | Procedure Description |
| --- | --- |
| FCB 00 | Suture of aortic arch |
| FCB 10 | Repair of aortic arch by division and suture |
| FCB 20 | Partial resection and suture of aortic arch |
| FCB 30 | Repair of aortic arch using patch |
| FCB 35 | Percutaneous insertion of stent into aortic arch  *Includes: Of plain mesh stent and coated stent (stentgraft)* |
| FCB 40 | Resection and reconstruction of aortic arch using tube graft |
| FCB 50 | Resection of aortic arch and reimplantation of branches  *Includes: Use of tube graft* |
| FCB 80 | Removal of foreign body from aortic arch |
| FCB 82 | Percutaneous removal of foreign body from aortic arch |
| FCB 96 | Other repair of aortic arch |

# **FMD Replacement of aortic valve**

| Code | Procedure Description |
| --- | --- |
| FMD30 | Replacement of aortic root using homograft and reimplantation of coronary arteries |
| FMD33 | Replacement of aortic root using xenograft and reimplantation of coronary arteries |

**Table S2.** **Distribution of surgical procedures over time for aortic aneurysm.** Arch surgery is defined as procedures involving reimplantation of arch branch vessels. Hemiarch replacement without branch vessel reimplantation is not classified as arch surgery.

| **Year** | **Total** | **Non-arch surgery** | **Arch surgery** |
| --- | --- | --- | --- |
| 2001 | 131 | 128 | 3 |
| 2002 | 197 | 189 | 8 |
| 2003 | 213 | 204 | 9 |
| 2004 | 158 | 153 | 5 |
| 2005 | 239 | 232 | 7 |
| 2006 | 242 | 237 | 5 |
| 2007 | 291 | 278 | 13 |
| 2008 | 283 | 275 | 8 |
| 2009 | 305 | 293 | 12 |
| 2010 | 325 | 310 | 15 |
| 2011 | 347 | 316 | 31 |
| 2012 | 352 | 335 | 17 |
| 2013 | 388 | 365 | 23 |
| 2014 | 380 | 351 | 29 |
| 2015 | 359 | 340 | 19 |
| 2016 | 382 | 364 | 18 |
| 2017 | 409 | 388 | 21 |
| 2018 | 486 | 466 | 20 |
| 2019 | 517 | 496 | 21 |
| 2020 | 425 | 404 | 21 |

**Table S3. Distribution of surgical procedures over time for aortic dissection.** Arch surgery is defined as procedures involving reimplantation of arch branch vessels. Hemiarch replacement without branch vessel reimplantation is not classified as arch surgery.

| **Year** | **Total** | **Non-arch surgery** | **Arch surgery** |
| --- | --- | --- | --- |
| 2001 | 107 | 101 | 6 |
| 2002 | 143 | 133 | 10 |
| 2003 | 135 | 128 | 7 |
| 2004 | 118 | 114 | 4 |
| 2005 | 141 | 131 | 10 |
| 2006 | 172 | 165 | 7 |
| 2007 | 163 | 157 | 6 |
| 2008 | 157 | 152 | 5 |
| 2009 | 179 | 173 | 6 |
| 2010 | 169 | 161 | 8 |
| 2011 | 177 | 163 | 14 |
| 2012 | 204 | 186 | 18 |
| 2013 | 193 | 184 | 9 |
| 2014 | 197 | 185 | 12 |
| 2015 | 219 | 195 | 24 |
| 2016 | 220 | 203 | 17 |
| 2017 | 205 | 187 | 18 |
| 2018 | 255 | 236 | 19 |
| 2019 | 267 | 245 | 22 |
| 2020 | 239 | 220 | 19 |

| **Table S4. Baseline characteristics and outcomes stratified by five-year intervals during the study period (surgery for aortic aneurysm, including both first-time operations and reoperations).** | | | | | |
| --- | --- | --- | --- | --- | --- |
| Time Period | **Overall**  n = 6,429 | **2001-2005**  n = 938 | **2006-2010**  n = 1,446 | **2011-2015**  n = 1,826 | **2016-2020**  n = 2,219 |
| Age (years), mean (SD) | 61·3 (12·8) | 60·2 (13·4) | 61·1 (12·4) | 61·5 (12·6) | 61·7 (13·0) |
| Age category (years), n (%) |  |  |  |  |  |
| ≤64 | 3,334 (51·9) | 527 (56·2) | 777 (53·7) | 931 (51·0) | 1,099 (49·5) |
| 65-74 | 2,288 (35·6) | 308 (32·8) | 487 (33·7) | 673 (36·9) | 820 (37·0) |
| ≥75 | 807 (12·6) | 103 (11·0) | 182 (12·6) | 222 (12·2) | 300 (13·5) |
| Sex, n (%) |  |  |  |  |  |
| Female | 1,870 (29·1) | 273 (29·1) | 417 (28·8) | 555 (30·4) | 625 (28·2) |
| Creatinine clearance (ml/min), mean (SD) | 95·7 (35·1) | 88·7 (34·2) | 96·4 (34·4) | 97·1 (36·0) | 96·9 (34·8) |
| Unknown, n | 399 | 87 | 161 | 104 | 47 |
| Diabetes, n (%) | 335 (5·3) | 29 (3·3) | 69 (5·0) | 99 (5·5) | 138 (6·2) |
| Unknown, n | 142 | 52 | 75 | 14 | 1 |
| CHA_2_DS_2_-VASc, mean (SD) | 2·7 (1·4) | 1·7 (1·2) | 2·3 (1·2) | 2·7 (1·2) | 3·4 (1·5) |
| 30-day mortality (first-time operations only), n (%) | 143 (2·4) | 35 (3·9) | 30 (2·2) | 42 (2·5) | 36 (1·8) |
| Perioperative stroke, n (%) | 221 (3·5) | 45 (5·1) | 30 (2·1) | 60 (3·3) | 86 (3·9) |
| Unknown, n | 85 | 54 | 24 | 6 | 1 |

| **Table S5. Baseline characteristics and outcomes stratified by 5-year intervals during the study period (surgery for aortic dissection, including both first-time operations and reoperations).** | | | | | |
| --- | --- | --- | --- | --- | --- |
| Time Period | **Overall**  n = 3,660 | **2001-2005**  n = 644 | **2006-2010**  n = 840 | **2011-2015**  n = 990 | **2016-2020**  n = 1,186 |
| Age (years), mean (SD) | 62·1 (11·9) | 60·8 (12·0) | 61·0 (11·6) | 62·2 (12·1) | 63·5 (11·7) |
| Age category (years), n (%) |  |  |  |  |  |
| ≤64 | 1,928 (52·7) | 375 (58·2) | 487 (58·0) | 505 (51·0) | 561 (47·3) |
| 65-74 | 1,202 (32·8) | 184 (28·6) | 248 (29·5) | 350 (35·4) | 420 (35·4) |
| ≥75 | 530 (14·5) | 85 (13·2) | 105 (12·5) | 135 (13·6) | 205 (17·3) |
| Sex, n (%) |  |  |  |  |  |
| Female | 1,221 (33·4) | 190 (29·5) | 294 (35·0) | 338 (34·1) | 399 (33·6) |
| Creatinine clearance (ml/min), mean (SD) | 85·9 (34·8) | 76·7 (30·8) | 87·3 (36·7) | 88·5 (34·6) | 86·5 (34·8) |
| Unknown, n | 693 | 247 | 233 | 155 | 58 |
| Diabetes, n (%) | 142 (4·1) | 17 (2·8) | 25 (3·4) | 49 (5·0) | 51 (4·3) |
| Unknown, n | 167 | 35 | 110 | 15 | 7 |
| CHA_2_DS_2_-VASc, mean (SD) | 2·6 (1·5) | 1·5 (1·1) | 2·1 (1·2) | 2·8 (1·3) | 3·5 (1·5) |
| 30-day mortality (first-time operations only), n (%) | 491 (14·6) | 126 (20·5) | 121 (15·5) | 114 (12·8) | 130 (12·0) |
| Perioperative stroke, n (%) | 492 (13·9) | 88 (15·1) | 110 (13·7) | 127 (13·0) | 167 (14·1) |
| Unknown, n | 111 | 62 | 35 | 11 | 3 |

**Table S6. Odds ratio for 30-days mortality after aneurysm surgery, first-time operations, reference year 2001.** Adjusted for age and, when applicable, sex. OR computed from generalized additive models, confidence intervals adjusted for simultaneous inference using Dunnett’s method.

| **Year** | **Male** | **Female** | **All** |
| --- | --- | --- | --- |
| 2001 | 1·00 (1·00–1·00) | 1·00 (1·00–1·00) | 1·00 (1·00–1·00) |
| 2002 | 0·94 (0·89–0·99) | 0·96 (0·89–1·03) | 0·94 (0·90–0·99) |
| 2003 | 0·88 (0·78–0·98) | 0·91 (0·79–1·06) | 0·89 (0·81–0·97) |
| 2004 | 0·82 (0·70–0·97) | 0·88 (0·70–1·10) | 0·84 (0·73–0·96) |
| 2005 | 0·77 (0·62–0·96) | 0·84 (0·62–1·13) | 0·79 (0·66–0·95) |
| 2006 | 0·72 (0·55–0·95) | 0·80 (0·55–1·17) | 0·75 (0·60–0·93) |
| 2007 | 0·67 (0·48–0·94) | 0·77 (0·49–1·20) | 0·71 (0·54–0·92) |
| 2008 | 0·63 (0·43–0·93) | 0·73 (0·43–1·24) | 0·67 (0·49–0·91) |
| 2009 | 0·59 (0·38–0·92) | 0·70 (0·38–1·28) | 0·63 (0·44–0·90) |
| 2010 | 0·55 (0·34–0·91) | 0·67 (0·34–1·32) | 0·59 (0·40–0·88) |
| 2011 | 0·52 (0·30–0·90) | 0·64 (0·30–1·36) | 0·56 (0·36–0·87) |
| 2012 | 0·49 (0·26–0·89) | 0·61 (0·27–1·40) | 0·53 (0·32–0·86) |
| 2013 | 0·45 (0·23–0·88) | 0·59 (0·24–1·44) | 0·50 (0·29–0·85) |
| 2014 | 0·43 (0·21–0·87) | 0·56 (0·21–1·49) | 0·47 (0·26–0·84) |
| 2015 | 0·40 (0·18–0·87) | 0·54 (0·19–1·53) | 0·44 (0·24–0·83) |
| 2016 | 0·37 (0·16–0·86) | 0·51 (0·17–1·58) | 0·42 (0·21–0·82) |
| 2017 | 0·35 (0·14–0·85) | 0·49 (0·15–1·63) | 0·39 (0·19–0·80) |
| 2018 | 0·33 (0·13–0·84) | 0·47 (0·13–1·68) | 0·37 (0·17–0·79) |
| 2019 | 0·31 (0·11–0·83) | 0·45 (0·12–1·73) | 0·35 (0·16–0·78) |
| 2020 | 0·29 (0·10–0·82) | 0·43 (0·10–1·79) | 0·33 (0·14–0·77) |

**Table S7. Odds ratio for 30-days mortality after dissection surgery, first-time operations, reference year 2001.** Adjusted for age and, when applicable, sex. OR computed from generalized additive models, confidence intervals adjusted for simultaneous inference using Dunnett’s method.

| **Year** | **Male** | **Female** | **All** |
| --- | --- | --- | --- |
| 2001 | 1·00 (1·00–1·00) | 1·00 (1·00–1·00) | 1·00 (1·00–1·00) |
| 2002 | 0·93 (0·83–1·05) | 0·94 (0·64–1·39) | 0·95 (0·93–0·98) |
| 2003 | 0·87 (0·69–1·08) | 0·89 (0·45–1·76) | 0·91 (0·86–0·96) |
| 2004 | 0·80 (0·59–1·09) | 0·83 (0·35–1·98) | 0·86 (0·80–0·93) |
| 2005 | 0·75 (0·52–1·08) | 0·77 (0·29–2·06) | 0·82 (0·74–0·91) |
| 2006 | 0·69 (0·45–1·06) | 0·72 (0·25–2·02) | 0·78 (0·69–0·89) |
| 2007 | 0·64 (0·40–1·02) | 0·67 (0·23–1·93) | 0·75 (0·64–0·87) |
| 2008 | 0·59 (0·36–0·97) | 0·64 (0·22–1·84) | 0·71 (0·59–0·85) |
| 2009 | 0·55 (0·33–0·92) | 0·63 (0·22–1·80) | 0·68 (0·55–0·83) |
| 2010 | 0·51 (0·30–0·86) | 0·64 (0·23–1·81) | 0·65 (0·51–0·81) |
| 2011 | 0·47 (0·28–0·81) | 0·68 (0·24–1·88) | 0·62 (0·48–0·79) |
| 2012 | 0·44 (0·26–0·76) | 0·72 (0·26–1·98) | 0·59 (0·44–0·78) |
| 2013 | 0·42 (0·24–0·72) | 0·77 (0·28–2·09) | 0·56 (0·41–0·76) |
| 2014 | 0·40 (0·23–0·69) | 0·81 (0·30–2·18) | 0·53 (0·38–0·74) |
| 2015 | 0·38 (0·22–0·66) | 0·83 (0·31–2·22) | 0·51 (0·36–0·72) |
| 2016 | 0·37 (0·21–0·64) | 0·80 (0·30–2·15) | 0·48 (0·33–0·71) |
| 2017 | 0·36 (0·21–0·62) | 0·72 (0·27–1·93) | 0·46 (0·31–0·69) |
| 2018 | 0·35 (0·20–0·62) | 0·60 (0·22–1·63) | 0·44 (0·28–0·67) |
| 2019 | 0·34 (0·19–0·62) | 0·48 (0·17–1·36) | 0·42 (0·26–0·66) |
| 2020 | 0·33 (0·17–0·63) | 0·37 (0·11–1·20) | 0·40 (0·25–0·64) |

**Table S8. Odds ratio for perioperative stroke after aneurysm surgery, first-time operations, reference year 2001.** Adjusted for age and, when applicable, sex. OR computed from generalized additive models, confidence intervals adjusted for simultaneous inference using Dunnett’s method.

| **Year** | **Male** | **Female** | **All** |
| --- | --- | --- | --- |
| 2001 | 1·00 (1·00–1·00) | 1·00 (1·00–1·00) | 1·00 (1·00–1·00) |
| 2002 | 0·86 (0·65–1·13) | 0·73 (0·43–1·22) | 0·76 (0·54–1·05) |
| 2003 | 0·74 (0·45–1·23) | 0·54 (0·22–1·34) | 0·58 (0·33–1·04) |
| 2004 | 0·65 (0·33–1·29) | 0·41 (0·13–1·29) | 0·47 (0·23–0·98) |
| 2005 | 0·59 (0·26–1·32) | 0·32 (0·09–1·17) | 0·39 (0·17–0·89) |
| 2006 | 0·54 (0·22–1·33) | 0·26 (0·07–1·02) | 0·34 (0·14–0·81) |
| 2007 | 0·51 (0·19–1·32) | 0·22 (0·06–0·91) | 0·30 (0·13–0·74) |
| 2008 | 0·49 (0·18–1·33) | 0·20 (0·05–0·83) | 0·29 (0·12–0·70) |
| 2009 | 0·49 (0·18–1·34) | 0·20 (0·05–0·80) | 0·29 (0·12–0·70) |
| 2010 | 0·50 (0·18–1·38) | 0·21 (0·05–0·81) | 0·31 (0·13–0·73) |
| 2011 | 0·52 (0·19–1·43) | 0·23 (0·06–0·86) | 0·33 (0·14–0·77) |
| 2012 | 0·54 (0·20–1·48) | 0·25 (0·07–0·92) | 0·36 (0·16–0·82) |
| 2013 | 0·56 (0·21–1·53) | 0·27 (0·08–0·97) | 0·38 (0·17–0·86) |
| 2014 | 0·58 (0·22–1·57) | 0·29 (0·08–1·02) | 0·40 (0·18–0·89) |
| 2015 | 0·61 (0·23–1·61) | 0·30 (0·09–1·05) | 0·41 (0·18–0·91) |
| 2016 | 0·63 (0·24–1·66) | 0·30 (0·08–1·04) | 0·42 (0·19–0·93) |
| 2017 | 0·66 (0·26–1·72) | 0·28 (0·08–0·97) | 0·42 (0·19–0·93) |
| 2018 | 0·70 (0·27–1·82) | 0·24 (0·07–0·86) | 0·42 (0·19–0·94) |
| 2019 | 0·75 (0·28–1·98) | 0·19 (0·05–0·78) | 0·42 (0·19–0·96) |
| 2020 | 0·80 (0·28–2·27) | 0·15 (0·03–0·79) | 0·42 (0·17–1·06) |

**Table S9. Odds ratio for perioperative stroke after dissection surgery, first-time operations, reference year 2001.** Adjusted for age and, when applicable, sex. OR computed from generalized additive models, confidence intervals adjusted for simultaneous inference using Dunnett’s method.

| **Year** | **Male** | **Female** | **All** |
| --- | --- | --- | --- |
| 2001 | 1·00 (1·00–1·00) | 1·00 (1·00–1·00) | 1·00 (1·00–1·00) |
| 2002 | 1·00 (0·95–1·05) | 1·00 (0·96–1·05) | 1·00 (0·97–1·03) |
| 2003 | 1·00 (0·90–1·11) | 1·00 (0·91–1·10) | 1·00 (0·94–1·07) |
| 2004 | 1·00 (0·86–1·16) | 1·01 (0·87–1·16) | 1·01 (0·92–1·10) |
| 2005 | 1·00 (0·83–1·21) | 1·01 (0·84–1·21) | 1·01 (0·89–1·14) |
| 2006 | 1·00 (0·80–1·26) | 1·01 (0·80–1·27) | 1·01 (0·87–1·17) |
| 2007 | 1·01 (0·77–1·31) | 1·01 (0·76–1·34) | 1·01 (0·85–1·21) |
| 2008 | 1·01 (0·75–1·35) | 1·01 (0·73–1·40) | 1·01 (0·83–1·24) |
| 2009 | 1·01 (0·73–1·40) | 1·01 (0·70–1·47) | 1·02 (0·81–1·28) |
| 2010 | 1·02 (0·71–1·44) | 1·02 (0·67–1·54) | 1·02 (0·79–1·31) |
| 2011 | 1·02 (0·70–1·48) | 1·02 (0·64–1·62) | 1·02 (0·77–1·35) |
| 2012 | 1·02 (0·69–1·53) | 1·02 (0·61–1·70) | 1·02 (0·76–1·38) |
| 2013 | 1·03 (0·67–1·57) | 1·02 (0·58–1·79) | 1·03 (0·74–1·42) |
| 2014 | 1·03 (0·66–1·61) | 1·02 (0·56–1·87) | 1·03 (0·73–1·46) |
| 2015 | 1·04 (0·65–1·66) | 1·03 (0·53–1·97) | 1·03 (0·71–1·49) |
| 2016 | 1·05 (0·64–1·71) | 1·03 (0·51–2·06) | 1·04 (0·70–1·54) |
| 2017 | 1·06 (0·63–1·77) | 1·03 (0·49–2·17) | 1·04 (0·69–1·58) |
| 2018 | 1·07 (0·62–1·83) | 1·03 (0·47–2·27) | 1·04 (0·67–1·62) |
| 2019 | 1·08 (0·61–1·90) | 1·03 (0·45–2·39) | 1·05 (0·66–1·67) |
| 2020 | 1·08 (0·60–1·97) | 1·03 (0·43–2·50) | 1·05 (0·64–1·72) |

**Table S10. Hospital length of stay after aortic aneurysm surgery, calculated from operation date to discharge from the cardiac surgery department** (missing values indicate unavailable discharge dates)

| **Time period** | **Total**  *n* | **Missing**  **discharge date**  *n* | **Median stay** (days) | **IQR** (days) | **Mean stay** (days) | **SD** (days) |
| --- | --- | --- | --- | --- | --- | --- |
| 2001-2005 | 938 | 24 | 7.0 | 4.0 | 8.8 | 6.1 |
| 2006-2010 | 1446 | 109 | 7.0 | 3.0 | 8.5 | 5.8 |
| 2011-2015 | 1826 | 82 | 6.0 | 3.0 | 7.8 | 6.3 |
| 2016-2020 | 2219 | 6 | 6.0 | 4.0 | 7.4 | 6.6 |

**Table S11. Hospital length of stay after aortic dissection surgery, calculated from operation date to discharge from the cardiac surgery department** (missing values indicate unavailable discharge dates)

| **Time period** | **Total**  *n* | **Missing**  **discharge date**  *n* | **Median stay** (days) | **IQR** (days) | **Mean stay** (days) | **SD** (days) |
| --- | --- | --- | --- | --- | --- | --- |
| 2001-2005 | 644 | 27 | 9.0 | 7.0 | 11.6 | 17.0 |
| 2006-2010 | 840 | 83 | 9.0 | 7.0 | 12.3 | 27.1 |
| 2011-2015 | 990 | 63 | 8.0 | 6.0 | 11.3 | 24.1 |
| 2016-2020 | 1186 | 11 | 8.0 | 6.0 | 10.7 | 14.2 |

**Supplementary figures**

**Figure S1.** **Modelled mean age at surgery for aneurysm and dissection from 2001 to 2020 estimated using generalized additive models, with 95% confidence bands.**


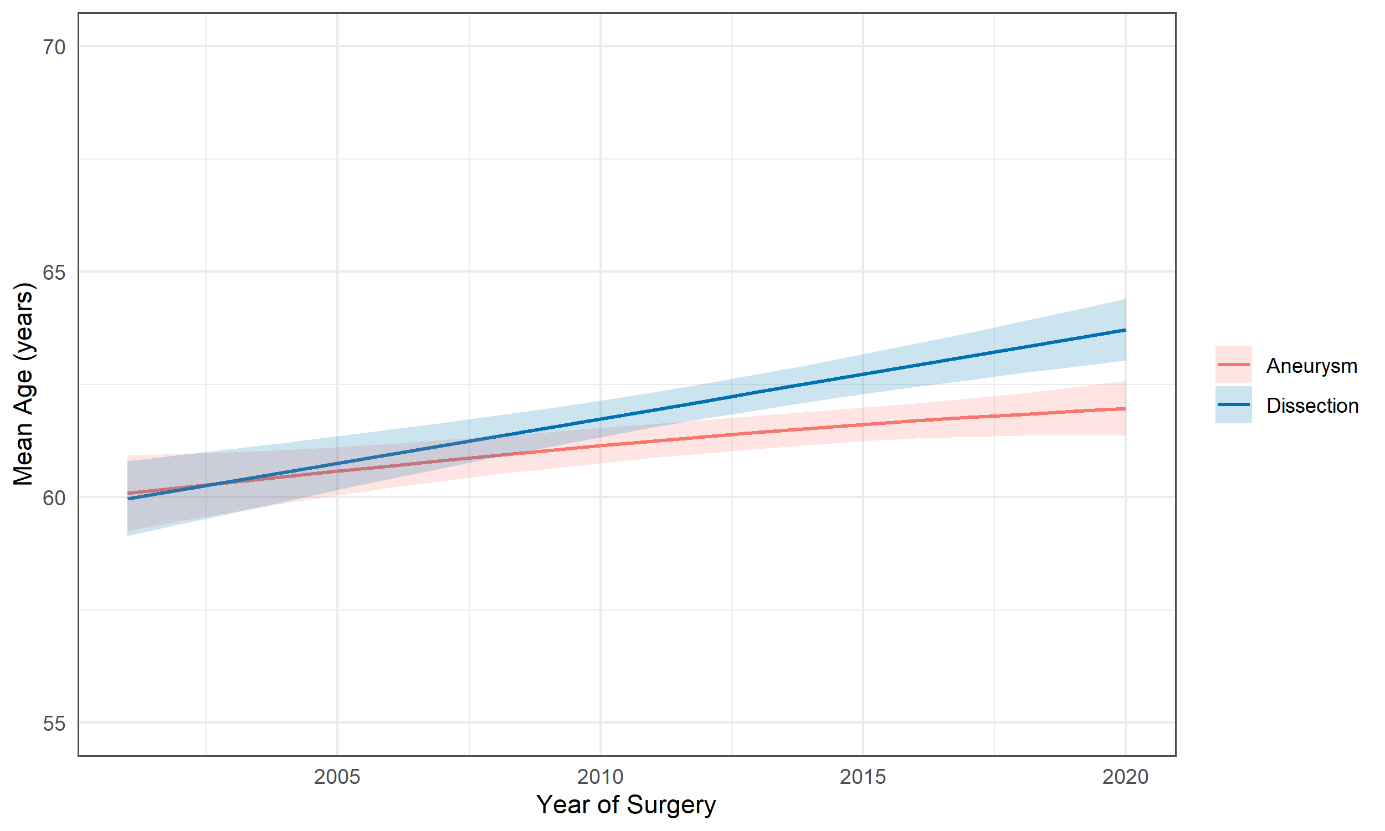


**Figure S2. Crude incidence of mortality and perioperative stroke in patients operated for aortic aneurysm or aortic dissection in different age groups.**


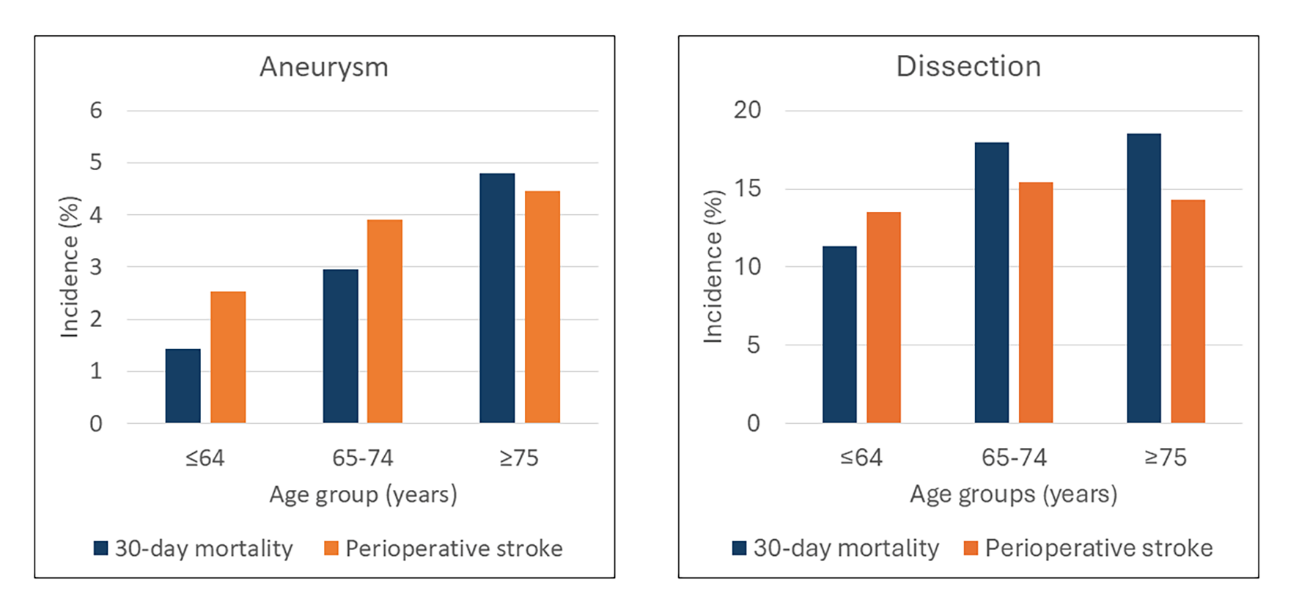


**Figure S3. Odds ratios for 30-day mortality and perioperative stroke from generalized additive models, stratified by diagnosis (aneurysm or dissection) and age, and adjusted for sex.** First-time operations only. Shaded areas indicate 95% confidence intervals. Reference year: 2001 (dotted line). aOR = adjusted odds ratio.


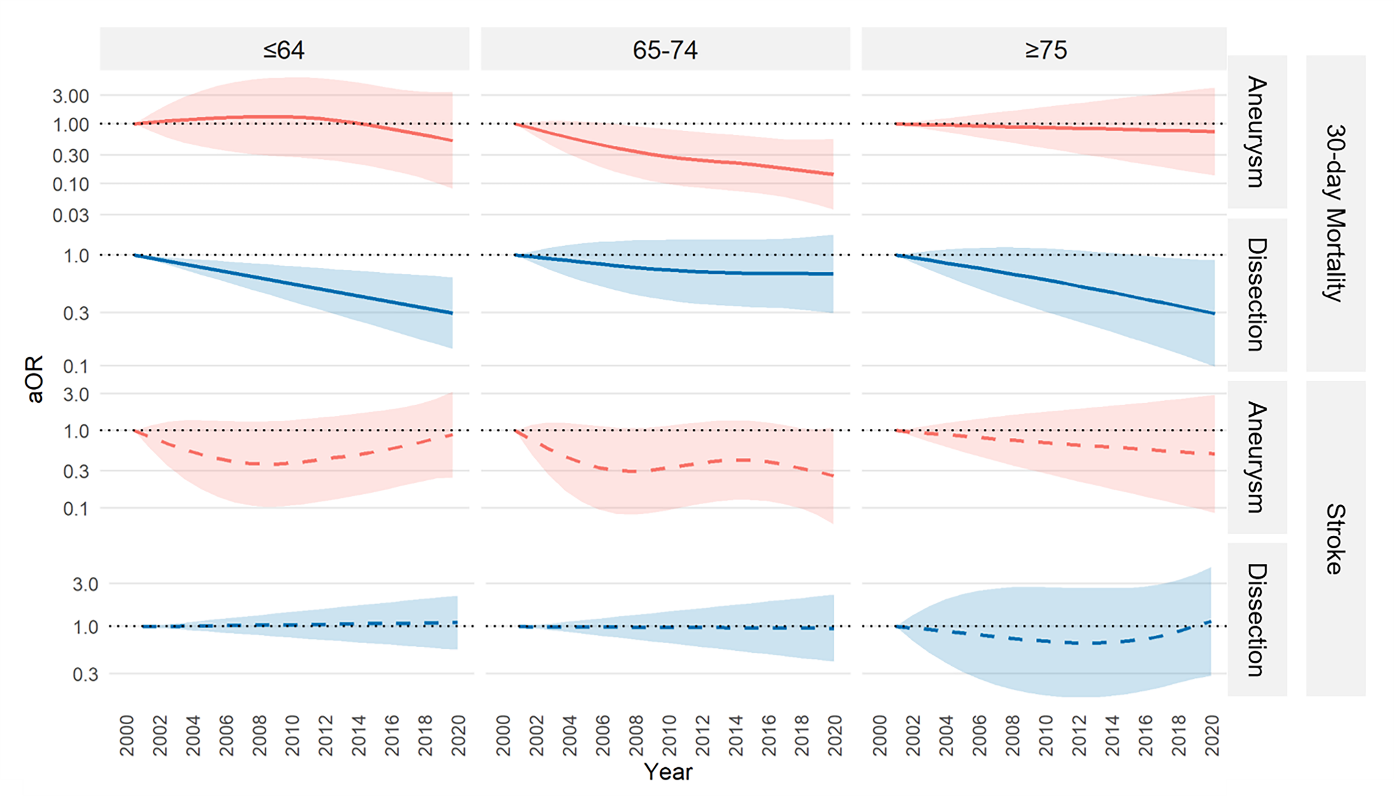


**Figure S4. Sensitivity analysis excluding 2020, the first year of COVID-19 pandemic.** **Odds ratios for 30-day mortality and perioperative stroke from generalized additive models, stratified by diagnosis (aneurysm or dissection) and sex, and adjusted for age and sex where applicable.** First-time operations only. Shaded areas indicate 95% confidence intervals. Reference year: 2001 (dotted line). aOR = adjusted odds ratio.


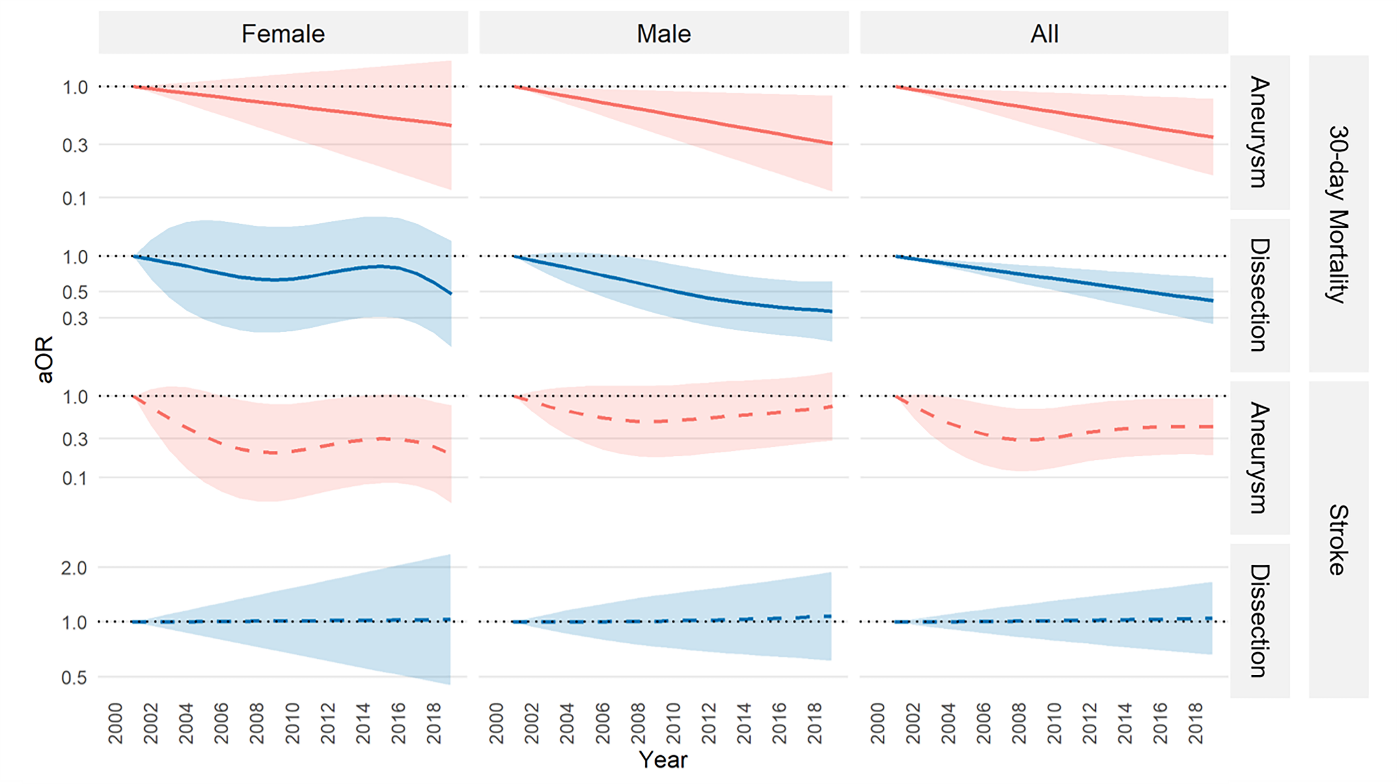

Supplement: Supplement Final [file mmc1.docx]
